# Solitary fibrous tumour of the mesentery: a case report

**DOI:** 10.1259/bjrcr.20170057

**Published:** 2017-10-04

**Authors:** Stephanie Vella, Christine Cannataci, Kelvin Cortis

**Affiliations:** ^1^Department of Medicine, Mater Dei Hospital, Msida, Malta; ^2^Medical Imaging Department, Mater Dei Hospital, Msida, Malta

## Abstract

We present the case of a male with an asymptomatic abdominal mass, where imaging-guided biopsy confirmed the lesion as a solitary fibrous tumour arising from the mesentery. This is a notably rare location for solitary fibrous tumours with only a few reported cases in the literature.

## Clinical presentation and imaging findings

A 71-year-old male was referred by his general practitioner in view of a palpable pulsatile lower abdominal mass noted on routine examination. The patient denied abdominal pain, weight loss, fever, change in appetite or bowel habit and passage of blood per rectum.

**Figure 1.**
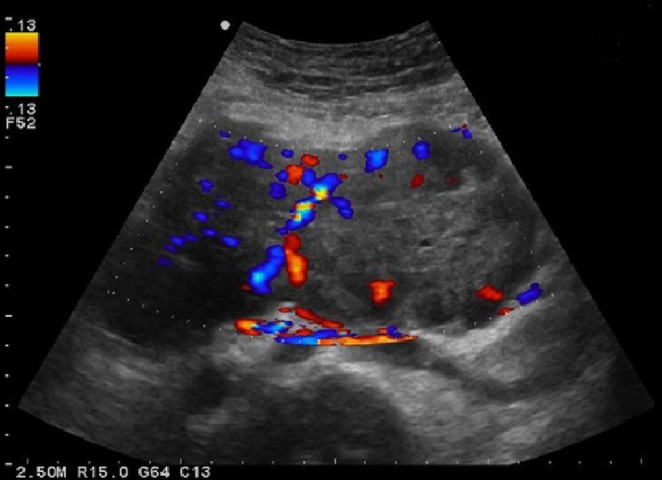
Ultrsound Doppler demonstrating hypervascularity of the mesenteric 15 × 7.2 × 13 cm mass.

**Figure 2.**
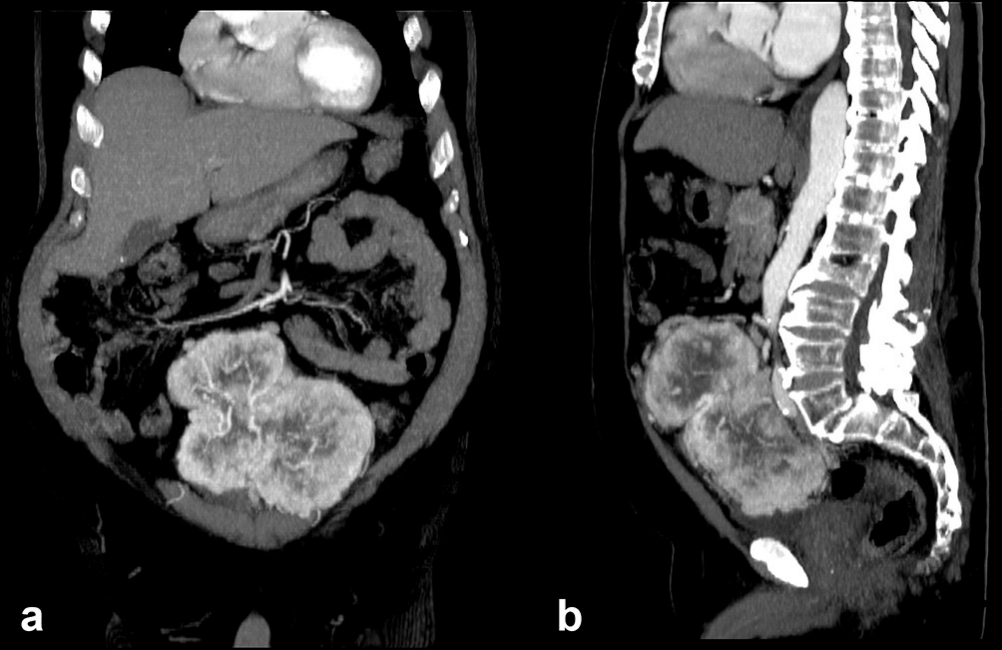
Enhanced arterial-phase CT demonstrates a multilobulated, well-defined, highly vascularized large mass situated at the mesentery with several areas of hypodensity corresponding with central necrosis.

An abdominal ultrasound was performed, which demonstrated a large heterogeneous, significantly vascularized mass anterior to the lower aorta (Figure 1). This was initially suspected to be an atypical lymphoma. Subsequent CT imaging showed a multilobulated well-defined highly vascularized large mass situated at the mesentery, about 15.0 cm in its largest diameter with multiple areas of low attenuation in keeping with central necrosis. This large mass was being supplied by multiple branches of the inferior mesenteric artery (Figure 2).

**Figure 3.**
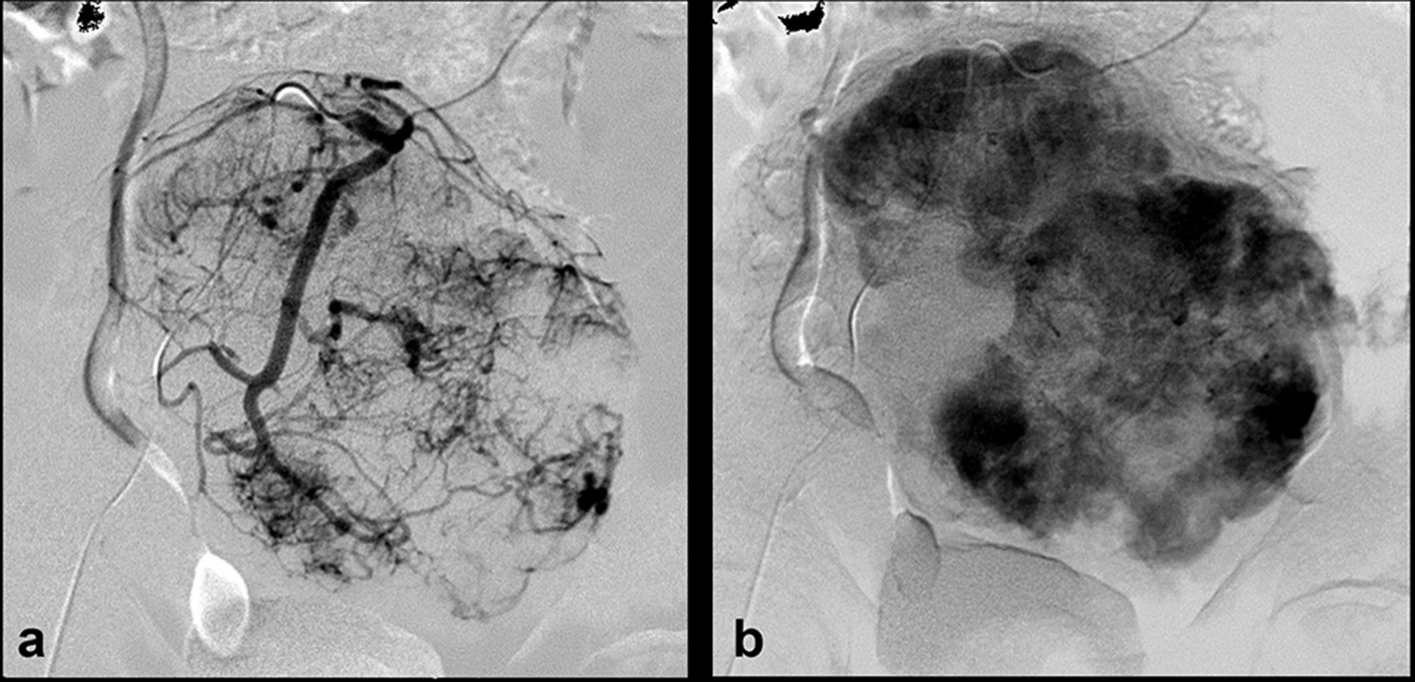
Direct invasive angiography with SOS2 catheter in the inferior mesenteric artery. Panel a: angiogram demonstrates highly vascularized lesion with multiple areas of neoangiogenesis. Panel b: delayed angiogram shows enhancement of the lesion.

An ultrasound-guided biopsy was performed and histopathology confirmed the mass to be a solitary fibrous tumour with no features of malignancy. The patient was admitted for two separate sessions of transarterial embolization of the tumour (Figure 3) in an attempt to reduce its size and vascularity prior to open surgical resection. The procedure was successful and the patient made a good clinical recovery. He was planned for routine imaging follow-up.

## Discussion

Solitary fibrous tumours (SFTs), first described in 1931, are rare mesenchymal tumours of fibroblastic or myofibroblastic origin (the exact origin is debatable from a histological standpoint).^1,2^ They comprise less than 2% of all soft tissue neoplasms, usually occur between the 4th and 6th decade of life and show no sex or ethnic predominance.^3^ Clinical presentation varies according to the site of involvement and includes pain, an asymptomatic palpable mass and symptoms secondary to pressure effects. A rare but well-described phenomenon is the occurrence of hypoglycaemia secondary to the production of insulin-like growth factor by some SFTs, known as Doege–Potter syndrome.^4^

Although SFTs were initially thought to only involve the pleura, a higher incidence of extrapleural SFTs is now recognised. In fact, SFTs have been found to arise from a myriad of sites including somatic structures (soft tissues and bone), head and neck (salivary gland, orbits, thyroid gland, meninges), as well as abdominal and pelvic structures (seminal vesicles, urinary bladder, prostate, liver, pancreas, kidney, retroperitoneum, gastrointestinal tract and mesentery).^5–7^ The mesentery is a very rare location for SFTs, with only a few cases being reported in the literature.^8^

While the majority of SFTs are benign, all have malignant potential. Indicators of malignancy include large tumour size (>10 cm) and histological findings such as high mitotic index, necrosis, cellular atypia and hypercellularity.^2^ However, even SFTs originally deemed histologically benign have demonstrated recurrence and metastasis after several years.^9^ The behaviour of malignant SFTs is closely related to mesothelial tumours with spread via seeding. In a study, metastases occurred in the liver, lung, mediastinum, peritoneum, omentum, colonic mesentery and subcutaneous tissue. According to some studies, extrathoracic SFTs seem to be more indolent than their intrathoracic counterparts with regard to metastasis; however, they confer a higher risk of local recurrence. Overall, these two classes are considered to be a single disease entity.^3,10^

Imaging features of SFTs have been described; though these are not entirely specific and vary depending on the tumour cellularity, vascularity and stromal density. Suggestive features on CT include a well-defined, hypervascular mass, with possible pressure effects on adjacent structures, subject to tumour size and location. In our case, the mass demonstrated organised hypervascularity with large feeding arteries and draining veins. Small masses are typically heterogeneous; however, in larger masses areas of central hypodensity may be present indicating necrosis or cystic change. While it has been reported that hypodense areas within the tumour may indicate the presence of malignancy, there are no pathognomonic CT features to confirm this.^11^ SFTs are usually isointense on *T*_1_ weighed MRI and isointense or hypointense on *T*_2 _weighting owing to the presence of fibrous tissue, with intense enhancement following gadolinium administration.^12^ Positron-emission tomography with fludeoxyglucose has shown accuracy in distinguishing SFTs from malignant disease. SFTs typically show a low level of fludeoxyglucose uptake (SUV < 2.5) if at all.^13,14^

 Differential diagnoses to be considered in our case include lymphoma, primary adenocarcinoma of the mesentery, mesenteric fibromatosis, inflammatory pseudotumour, solitary vascular metastatic lesion and extra-adrenal phaeochromocytoma of the organ of Zuckerkandl. In the case of lymphoma, the mass typically appears very homogeneous on CT (heterogeneous density is seen in very aggressive subtypes) with minimal to no enhancement due to the poor vascularity of the lesion. The presence of large collateral feeding vessels may be a useful imaging feature to distinguish SFTs from other masses; however, this is not specific.^15,16^

Treatment of SFTs involves *en bloc* surgical resection with negative margins. The role of neoadjuvant chemo- or radiotherapy is controversial, producing mixed results.^17^ Antiangiogenic therapy for unresectable SFTs may be promising.^18^

The prognosis of these tumours is not well defined; however, the high recurrence rate and the variability at histology necessitate long-term follow-up.^18,19^ There are no surveillance guidelines; though it is generally recommended to perform a CT scan every 6 months for the first 2 years and then annually.^20^

## Learning points

1. Solitary fibrous tumours (SFTs) are rare mesenchymal tumours comprising less than 2% of all soft tissue neoplasms.
2. While SFTs were initially thought to only involve the pleura, a higher incidence of extrapleural SFTs is now recognised.
3. Despite the large size of SFTs, patients are often asymptomatic at the time of diagnosis.
4. Management involves surgical resection with long-term follow-up.

## Consent

Written informed consent for the case to be published (including images, case history and data) was obtained from the patient(s) for publication of this case report, including accompanying images.
